# NMDA Receptor Subunits Change after Synaptic Plasticity Induction and Learning and Memory Acquisition

**DOI:** 10.1155/2018/5093048

**Published:** 2018-03-07

**Authors:** María Verónica Baez, Magalí Cecilia Cercato, Diana Alicia Jerusalinsky

**Affiliations:** Instituto de Biología Celular y Neurociencia “Prof. E. De Robertis”, UBA-CONICET, School of Medicine, University of Buenos Aires, 2155 Paraguay St., 1121 CABA, Argentina

## Abstract

NMDA ionotropic glutamate receptors (NMDARs) are crucial in activity-dependent synaptic changes and in learning and memory. NMDARs are composed of two GluN1 essential subunits and two regulatory subunits which define their pharmacological and physiological profile. In CNS structures involved in cognitive functions as the hippocampus and prefrontal cortex, GluN2A and GluN2B are major regulatory subunits; their expression is dynamic and tightly regulated, but little is known about specific changes after plasticity induction or memory acquisition. Data strongly suggest that following appropriate stimulation, there is a rapid increase in surface GluN2A-NMDAR at the postsynapses, attributed to lateral receptor mobilization from adjacent locations. Whenever synaptic plasticity is induced or memory is consolidated, more GluN2A-NMDARs are assembled likely using GluN2A from a local translation and GluN1 from local ER. Later on, NMDARs are mobilized from other pools, and there are de novo syntheses at the neuron soma. Changes in GluN1 or NMDAR levels induced by synaptic plasticity and by spatial memory formation seem to occur in different waves of NMDAR transport/expression/degradation, with a net increase at the postsynaptic side and a rise in expression at both the spine and neuronal soma. This review aims to put together that information and the proposed hypotheses.

## 1. Introduction

Learning and memory, as well as synaptic plasticity which is considered their electrophysiological correlate, depend on glutamatergic transmission (reviewed in [1]). AMPA (*α*-amino-3-hydroxy-5-methyl-4-isoxazolepropionic acid) and NMDA (*N*-methyl-D-aspartate) ionotropic glutamate receptors (AMPARs and NMDARs, resp.) are crucial in activity-dependent synaptic changes (reviewed in [2–5]). While AMPARs are able to mediate ordinary glutamate neurotransmission, the NMDAR channel is usually blocked by extracellular Mg^2+^ at cell membrane resting potentials (reviewed in [1]). To achieve effective transmission through NMDARs, they must be unblocked by membrane depolarization through the activation of AMPARs, while at the same time, both glutamate and glycine must bind the receptor. Therefore, NMDARs are considered molecular coincidence detectors of pre- and postsynaptic activities [6].

NMDARs are heterotetramers mainly present not only at the postsynaptic but also at the presynaptic side of glutamatergic synapses in the central nervous system (CNS). NMDARs are composed of 2 GluN1 obligatory subunits encoded by one gene, with eight variants originated by alternative splicing [7, 8], and 2 regulatory subunits that contain the glutamate binding site. Those regulatory subunits are encoded by different genes; there are four GluN2 subunits (GluN2A–D), which are codified by four different genes, and two GluN3 subunits (GluN3A and B), which are codified by two different genes [5, 9]. At least theoretically, there are more than 60 possibilities to combine these subunits. However, only 9 receptor subtypes have been described, which could be classified as diheteromeric (GluN1_2_-GluN2A_2_, GluN1_2_-GluN2B_2_, GluN1_2_-GluN2C_2_, GluN1_2_-GluN2D_2_, and GluN1_2_-GluN3A_2_) and triheteromeric (GluN1_2_-GluN2A-GluN2B, GluN1_2_-GluN2B-GluN2C, GluN1_2_-GluN2B-GluN2D, and GluN1_2_-GluN2B-GluN3A) (reviewed in [5, 10, 11]).

The regulatory subunit composition of NMDARs defines its pharmacological and kinetic properties [3–5, 9, 12]. Expression of regulatory subunits is dynamic and seems to be tightly regulated in time and space [4, 12, 13]. In CNS regions involved in cognitive functions, like the hippocampus and prefrontal cortex (PFC), GluN2A and GluN2B are the major regulatory subunits [5, 13].

During prenatal life, NMDARs containing GluN2B subunit (GluN2B-NMDAR) are predominant, being GluN2B the major regulatory subunit expressed along embryonic development in studied mammals [14]. During early postnatal life, there is an increase in GluN2A expression both in transcription and translation, while GluN2B expression appears to remain low and constant. As a consequence, the GluN2A/GluN2B ratio rises up during that period [15, 16], known as the “NMDAR developmental switch” (reviewed in [12, 13]). In Sprague-Dawley rats, a further increase in GluN2A levels appears to occur after that developmental switch, at least in the hippocampus, where the GluN2A/GluN2B ratio was reported to be higher in hippocampal protein extracts obtained from 6-month-old rats compared to 35-day-old rats [14]. Although there are several reports, little is known about changes in NMDAR expression after plasticity induction or memory acquisition and the putative involved mechanisms and physiological meaning of such modifications. This review aims to put together that information and the proposed hypotheses on those changes.

## 2. Changes in NMDAR Expression after Plasticity Induction (Table 1)

NMDARs participate in physiological plasticity in the nervous system during development, as well as in synaptogenesis and synapse maturation along the whole life. Also, NMDARs are involved in pathological forms of plasticity, as in epilepsy (reviewed in [4]), stroke and hypoxia (reviewed in [17, 18]), and neurodegenerative disorders like Alzheimer's (reviewed in [5, 13, 19]) and Parkinson's disease (reviewed in [5, 13, 20, 21]). The lack of either NMDARs or some of their subtypes (i.e., knocking down either GluN1 expression or some regulatory subunit) led to different sorts of long-term plasticity (LTP) or depression (LTD) deficits (reviewed in [22–25]). NMDAR-dependent synaptic plasticity could also be affected by stress or corticosterone treatment ([26]), ethanol (reviewed in [27]), and several drug exposure (reviewed in [13]).

Williams et al. [28] had reported that there were several waves of NMDAR subunit rises, after LTP induction by high-frequency stimulation (HFS). Both GluN2A and GluN2B levels increased 20 minutes and 48 h after LTP induction in DG total extracts, without significant changes 1 h or 4 h after stimulation. Later on, it was shown that both GluN1 and GluN2B levels were significantly higher in synaptosomal fractions 20 min and 48 hours after HFS and that there also was an increase in GluN1 8 h after stimulation in total DG homogenates [29]. The authors attributed the 48 h rise in GluN1 mainly to an increase in NMDARs at the cell surface, though not at the synaptic membrane, as GluN1 was not significantly higher in the postsynaptic density fraction [30].

On the other hand, Bellone and Nicoll [31] have shown that LTP induction by HFS, in fresh hippocampal slices from newborn mice, led to a rapid shift from GluN2B-NMDAR to GluN2A-NMDAR mediated currents. Those changes take place in milliseconds to seconds and were attributed to simultaneous lateral mobilization of GluN2A-NMDAR along the membrane from extrasynaptic sites and internalization of GluN2B-NMDAR. Furthermore, Barria and Malinow [32] reported a GluN2A-NMDAR increase in dendritic spines after LTP induction in organotypic cultures of hippocampal slices from neonatal rats (immunocytochemistry). Accordingly, Grosshans et al. [33] showed that GluN1 and GluN2A levels were higher in western blots of synaptosomal fractions, correlated with lower levels in these same subunits in nonsynaptic fractions, 30 minutes after plasticity induction in hippocampal slices from 6- to 8-week-old rats. These works suggested strongly that the GluN2A-NMDAR rise at the synapses could be due to mobilization of preassembled NMDARs from nonsynaptic pools [32, 33].

We have analyzed NMDAR subunits level following induction of LTP by theta burst stimulation (TBS), in young adult rat hippocampus fresh slices. In slices where potentiation seemed to be effective up to 30 minutes post-TBS (at least as short-term potentiation), GluN1, GluN2A, and GluN2B total levels remained similar to control levels [34]. It has to be taken into account that, while Grosshans et al. determined NMDAR subunits in synaptosomes [35], we used total hippocampal homogenates [34], where exchanges between subcellular fractions could be masked.

We have also reported that 70 minutes after effective LTP induction by TBS, in fresh hippocampal slices from young adult rats, there was an increase in GluN1 and GluN2A levels, while GluN2B remained constant, as determined by western blot in total hippocampus extracts. The rise in both GluN1 and GluN2A subunits only occurred when an effective long-term synaptic plasticity (lasting more than 60 minutes) was established [34]. It must be emphasized that in TBS-stimulated slices where potentiation failed, GluN1, GluN2A, and GluN2B levels remained similar to controls (without stimulation) at 30 and 70 minutes after TBS.

In line with those results, Udagawa et al. [36] showed in primary neuron cultures stimulated by NMDA that GluN2A mRNA localizes in dendrites, where it is inefficiently translated due to its short poly(A) tail. However, after NMDAR stimulation, Gld2 (a poly(A) polymerase) catalyzes poly(A) addition to GluN2A mRNA, leading to translational enhancement of GluN2A mRNA and to an increase in GluN2A levels (measured by western blot), 30 minutes after NMDAR stimulation. The same authors reported that GluN1, but not GluN2B, also increased in dendrites at the same time as GluN2A, but independently of poly(A) polymerization. Furthermore, Swanger et al. [37] have shown that GluN2A local translation and surface expression initiate immediately after LTP induction and continue at least for the following 30 minutes (the last time point they analyzed).

In a similar model, we have found that there are equivalent changes in NMDAR subunits even after 30 min poststimulation [34, 38]. GluN2A *puncta* was significantly increased in cultured hippocampal neurons 30 minutes poststimulation by KCl pulses and it continued rising up to 75 minutes. Thereafter, GluN2A *puncta* decreases, reaching control levels 90 minutes after stimulation. On the other hand, GluN1 also starts to increase at 30 minutes, reaching a maximum at 75 minutes. GluN1, as GluN2A *puncta*, did not differ from nonstimulated controls 90 minutes poststimulus [38].

Some reports have suggested that NMDAR levels could be degraded by different mechanisms. For instance, the binding of GluN1 to Fbx2 (a F box protein associated to the E2 ligase complex), labels this particular NMDAR as proteasome target [39]. Nevertheless, further investigation is necessary in order to clarify NMDAR subunit degradation mechanism after plasticity induction. In line with this, Corbel et al. [40] have shown that GluN2A translation is regulated by miR19 along development, decreasing GluN2A expression during early development.

Concerning possible mechanisms involved in the rise of NMDAR subunits, CHX treatment either totally [37] or partially blocked [34, 38] the increase in GluN2A subunits at dendrites. However, this (CHX) treatment did not affect GluN1 increase at dendrites, suggesting that GluN2A-NMDAR increase could be due to local translation of GluN2A subunit, which would rapidly be assembled with GluN1 subunits retained inside some local ER vesicles [37, 38].

Furthermore, when CHX was previously added to the media, the increase of both GluN1 and GluN2A subunits, which achieves a maximum at 70–75 minutes after plasticity induction, was fully blocked in neuronal bodies (cultured neurons) as well as in hippocampal slice homogenates. On the other hand, actinomycin D (ActD) treatment did not affect either LTP induction in hippocampal slices or GluN2A increase in both slice homogenates and hippocampal neuron cultures. However, ActD treatment blocked GluN1 increase both in slice homogenates after LTP induction and in soma of cultured neurons after KCl stimulation, indicating that, at least for the GluN1 rise, de novo synthesis was necessary [34, 38].

**Figure 1:**
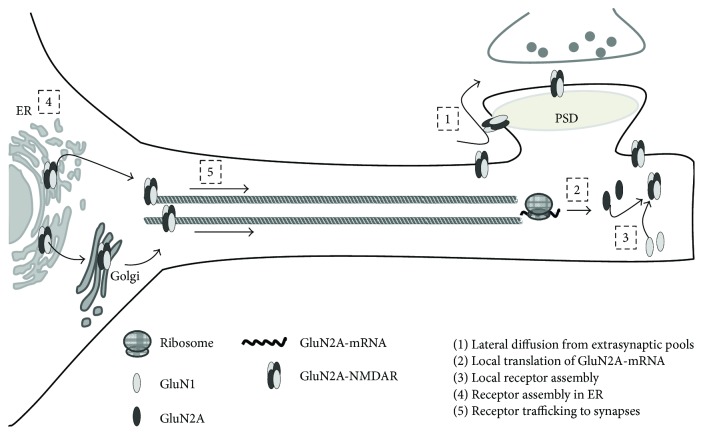
Schematic representation of the proposed model for NMDAR localization and expression after plasticity induction. After a stimulus that would elicit long-term plasticity, there is a rapid increase in surface GluN2A-NMDAR at the postsynaptic side, which is likely due to lateral receptor mobilization from adjacent locations (step 1). Whenever plasticity was effectively induced, more GluN2A-NMDARs would be assembled using GluN2A from local translation and GluN1 retained in local ER (step 2). As more NMDARs are needed, mobilization from other pools would contribute to enhance NMDAR expression at synapses (step 3). As nonsynaptic pools decrease, some signals should activate NMDAR subunits expression at the neuronal soma, which would lead to a transient increase in subunits level there (steps 4-5).

Altogether, the above-reported data strongly suggest that following an appropriate stimulus, there is a rapid increase in surface GluN2A-NMDAR at the postsynaptic side, which is likely due to lateral receptor mobilization from adjacent locations (Figure 1, step 1). Whenever plasticity was effectively induced, more GluN2A-NMDARs would be assembled using GluN2A from local translation and GluN1 retained in local ER (Figure 1, step 2). As more NMDARs are needed at the spines, mobilization from other pools would contribute to fill up these requirements (Figure 1, step 3). It is conceivable that when the different pools were decreasing, some signals activate NMDAR subunit expression at the neuronal soma, which would lead to a transient increase in subunit level there (Figure 1, steps 4-5). Once synaptic plasticity has been established and the postsynaptic side has already been remodeled, the high concentration of NMDARs could result in excitotoxicity. It is feasible that existent GluN2A-NMDAR would be regulated by ubiquitination and degradation by the proteasome and that de novo expression would be regulated, that is, by miRNAs [39, 40], restoring control levels.

## 3. NMDAR Expression and Memory Acquisition (Table 2)

NMDAR subunits increase was also reported to occur *in vivo* in animal models, after various experiences. Different changes in GluN1, GluN2A, and GluN2B have been reported to occur in several central structures (Table 2). Such differences could be due to the different techniques used to evaluate memory (including different tasks) and to determine NMDAR subunits. Here, we summarize main changes in NMDAR subunits levels after memory acquisition.

In a task classically used for spatial learning in the rat, the hidden platform version of the Morris water maze (MWM), Cavallaro et al. [41] have shown (by microarray followed by qPCR conformation) that GluN1 mRNA expression was downregulated 1 h after four consecutive training sessions, whereas GluN2A mRNA expression was similar to that in controls. They have also found that it was upregulated when assessed 24 h after training. However, in rats trained in the same task with a long-term memory paradigm, Zhang et al. [42] found that GluN1 immunofluorescence was increased in CA1 and DG after 10 trials. This last result is similar to that observed after synaptic plasticity induction described above in the previous section [34, 38]. Moreover, Zhang et al. have also shown that no significant difference was found in long-term memory expression for a long-trained (LT) group of animals compared to a short-trained with reinforcement group (SRT). Accordingly, the intensity of GluN1 immunoreactivity in CA1 and dentate gyrus in LT and SRT rats was significantly higher than that in short-trained or control groups. The comparison of both sets of results led to the interpretation that this increase of GluN1 expression in CA1 and dentate gyrus could be involved in spatial long-term memory formation.

We have shown that both GluN1 and GluN2A subunits increased in the hippocampus of 1-, 2-, and 3-month-old Wistar rats following habituation to a new environment (open field (OF) task) [34]. This increase begins after 30 minutes of a 5-minute session in an OF, which leads to habituation, and reaches a maximum at about 70 minutes. Thereafter, GluN1 and GluN2A levels fall down, being similar to controls at about 90 minutes posttraining. This time course was rather similar to that described *in vitro* for cultured hippocampal neurons [38]. Therefore, both *in vitro* and *in vivo* changes reported by others and us are transient, with rather similar time courses and direction. No significant changes were found, in other analyzed structures like amygdala and PFC 70 minutes after habituation to the OF, nor in the hippocampus after testing the rats in the OF 24 h later [34, 38]. Also, we described an increase in GluN1 and GluN2A subunits in the rat hippocampus with a similar time course, following the object exposure phase of a two-object recognition task, though not after the different tests; even when a familiar object and a new object were presented, the new object was effectively discriminated by the animal [38].

Interestingly, Hepp et al. [43] have recently found in an invertebrate that, although total GluN1 level remained unchanged in crabs after spatial memory acquisition, GluN1 expression at the neuron surface fell down immediately after a 50-minute training. The authors also assessed GluN1 surface expression 3 hours later and found that there was an increase in GluN1 in membrane samples. This increase was also transient, without difference with control level 24 h later.

NMDAR changes, like a GluN1 increase, were also reported following other spatial tasks like a radial maze and a hole board. Shanmugasundaram et al. [44] have observed, in a synaptosomal fraction extracted 6 h after training rats in a radial maze along 10 consecutive days, that there was a rise in both GluN1 and GluN2B at the hippocampus and an increase in GluN1 and GluN2A in PFC. The same team also found that there was an increase in GluN1 and GluN2A in the synaptosomal fraction of the dorsal hippocampus, and a later increase in GluN1 and GluN2B in PFC, in rats that were trained in a hole-board along 3 consecutive days and were then stimulated with a weak tetanizing stimulus that would not lead to late-LTP by itself, compared with nontrained though stimulated rats. In spite of the weak stimulation, in such trained rats, the “underthreshold” stimulus led to L-LTP that lasted up to 6 hours [45]. Unfortunately, in this case, it is not known if the raise in NMDAR subunits would take place anyway without the electrical stimulation.

A few works showed that there were changes in NMDAR expression and/or localization using paradigms that are associated with strong emotion. In a step-down inhibitory avoidance of a mild foot-electric shock, Cammarota et al. [46] showed that there was an increase of GluN1 in hippocampal synaptosomal fractions (by western blot), without significant changes in GluN2A or in GluN2B 30 minutes after training; 120 minutes after training, NMDAR subunits level was similar to controls. Mukherjee et al. [47, 48] showed that, in P7 to P10 pups, when the developmental switch from GluN2B to GluN2A did not take place yet [12], there was a change in NMDAR as the absolute amount of the essential GluN1 decreased three hours after one training session in an odor preference task. This decrease was observed in synaptosomal fractions of the anterior piriform cortex [47] and in postsynaptic density fractions [48]. However, 24 h after training, GluN1 level was not significantly different compared to controls, indicating that the modification was transient. Authors also showed that GluN1 downregulation was initiated by mGluR-mediated calcineurin signaling and inferred dephosphorylation and internalization of NMDARs. On the other hand, 24 h after two trials, there was no significant change in GluN1 level compared to control level.

Changes in NMDAR subunits level have also been described following fear conditioning, Sun et al. [49] have shown that there was a rapid and transient increase in the amount of membrane GluN2B-NMDARs (as both GluN1 and GluN2B increased) in CA1 area, 5 to 10 minutes after a single-trial fear conditioning training. The authors proposed that GluN1 and GluN2B increases depend on GluN2B activation, as it was blocked by GluN2B inhibitors. Accordingly, Sun et al. have also suggested that the reported subunit increase could depend on training strength, as a 5-trial conditioning induced higher subunits levels than a single-trial training [49].

## 4. Final Considerations on the Hypotheses Proposed

NMDAR's central role in synaptic plasticity under physiological conditions is based on its high permeability to calcium ions, as the triggering of both NMDAR-dependent LTP and LTD, at least in the CA1 region of the hippocampus, requires a rise in postsynaptic calcium. But this is also the basis of NMDAR's role in excitotoxic pathological conditions that could lead to neuronal death.

We wonder, for instance, how hippocampal neurons would avoid excitotoxicity after facilitation/potentiation induction through NMDARs, having into account the further increase they seem to undergo for long-term plasticity establishment and long-term memory consolidation. Since the cloning of the different subunits, searching the relationships between NMDAR subtypes and the corresponding functions has been a continuous challenge (reviewed in [4, 12, 13, 24]). As shown by pharmacological studies, activation of GluN2B-NMDARs led to excitotoxic cell death *in vitro* and *in vivo*, whereas activation of GluN2A-NMDARs appears neuroprotective [50]. Moreover, GluN2B-NMDARs are associated with pro-death cellular pathways [51, 52].

Could an increase in GluN2A, that is, at hippocampal synapses, represent a homeostatic mechanism to normalize synaptic plasticity modifications as to avoid unwanted collateral effects following long-term plasticity?

Altogether, these data could lead to the hypothesis that changes in GluN1 or NMDAR levels induced by synaptic plasticity and by, mainly, spatial memory formation seem to occur in different waves of NMDAR transport/expression/degradation, with an increase in postsynaptic membranes, a rise in local and central expression, followed by degradation and relocalization, and a decrease in expression. This waves could subserve to different synaptic/neuronal functions, depending on the structure, sign, and time period of the molecular/synaptic change.

We propose that the subunits increase from about 20–30 to 75 minutes, which has already disappeared at 90 minutes, after plasticity induction or memory acquisition, could be acting as a check point or a synaptic tag for plasticity establishment or memory consolidation. An increase in the synaptic GluN2A-NMDAR versus GluN2B-NMDAR ratio could act as stabilizer of some synaptic/circuital changes [51], hence leading to stabilize memory consolidation, particularly of spatial representations.

## Figures and Tables

**Table 1:** Changes in NMDAR subunits after plasticity induction. Changes in NMDAR expression or in NMDAR subunits level after inducing plasticity in several experimental models.

Animal model (age)	Experimental protocol	Structure analyzed	Method	Timing	NMDAR changes	Ref.
Adult Sprague-Dawley rats	LTP *in vivo* in the dentate gyrus	Dentate gyrus	WB of whole homogenates	0, 0.20, 1, 4, and 48 hs and 2 weeks after LTP induction	Increase of GluNB and GluN2A at 20 and 48 hs after LTP induction.	[28]
Sprague-Dawley rats (6–8 weeks)	LTP induction in CA1 minislices	Hippocampal CA1 region	(i) WB of synaptosomal membrane and light membrane fractions from minislices	30 min after LTP induction (subcellular fractionation assay)	Enhanced surface expression of GluN1 and GluN2A at 30 min after stimulation, with a significant decrease in the intracellular pools. There is no change in total NMDAR level (both methods).	[33]
			(ii) WB of BS3-treated homogenates (cross-linking assay) from minislices	0, 30, 60, 90, 120, 150, and 180 min (cross-linking assay)	The increase of GluN1 and GluN2A starts at 15 min and persists for at least 3 h after LTP induction (cross-linking assay).	
Rats (6-7 days)	LTP induction in organotypic cultures of hippocampal slices	Hippocampal CA1 region	Whole-cell recordings		GluN2A-NMDAR increase.	[32]
Adult Sprague-Dawley rats	LTP *in vivo* in the dentate gyrus	Dentate gyrus	WB of whole homogenates and synaptoneurosomes	0.20, 4, 8, and 48 h and 2 weeks after LTP induction (whole homogenates assay)	Increase of GluN1 at 8 and 48 h after LTP induction (whole homogenate assay).	[29]
				0.20 and 48 h (synaptoneurosomes assay)	Increase of GluN2B at 0.20 and 48 h following LTP induction. Increase of GluN1 at 48 h, but not at 20 min, post LTP induction (synaptoneurosomes assay).	
Sprague-Dawley rats (2 to 21 days)	LTP induction in hippocampal slices	Hippocampal CA1 region	NMDA EPSCs recordings	Milliseconds to seconds	Rapid synaptic shift from GluN2B-NMDAR to GluN2A-NMDAR in young, but not in adult, animals, after LTP induction.	[31]
Adult Sprague-Dawley rats	LTP *in vivo* in the dentate gyrus	Dentate gyrus	WB of synaptoneurosomes, biotin-tagged synaptic surface extracts and PSD fractions	20 h and 2 weeks after LTP induction	Increase of GluN1 in the surface-membrane fraction and synaptoneurosomes, but not in PSD fractions, at 48 h post LTP induction. GluN1 levels in surface extracts returning to baseline at 2 weeks post stimulation.	[30]
Sprague-Dawley rat embryos (E18)	Glycine stimulation in hippocampal neuron cultures (21–24 DIV)	Hippocampus	(i) WB of homogenates from biotinylated primary cultures (ii) IF of primary cultures	30 min after glycine stimulation	Enhanced surface expression of GluN1 and GluN2A after stimulation, without change in GluN2B. Glycine also increased total GluN1 and GluN2A protein levels.	[37]
Sprague-Dawley rat embryos (E18)	NMDA stimulation in hippocampal neuron cultures (21–24 DIV)	Hippocampus	(i) WB of homogenates from primary cultures (ii) IF of primary cultures	From immediately 30 min after stimulation	Enhanced surface expression of GluN1 and GluN2A after stimulation, without change in GluN2B. NMDA also increased total GluN1 and GluN2A protein levels.	[36]
Wistar rats (P 42–60)	LTP induction in hippocampal slices	Hippocampus	WB of whole-slice homogenates	0, 30, and 70 min after LTP induction	Increase of GluN1 and GluN2A, but not GluN2B, at 70 min after LTP induction.	[34]

**Table 2:** Changes in NMDAR subunits after memory acquisition. Changes in NMDAR subunits after training different animals in various behavioral paradigms.

Animal model (age)	Task	Structure analyzed	Method	Timing	NMDAR changes	Ref.
Domestic chicks (one day)	Passive avoidance	Forebrain	Quantitative autoradiography of ligand-receptor binding	30 min after training	Increased binding of glutamate and MK-801 tritiated to NMDA receptors.	[53]
Long Evans rats raised in complete darkness (21–23 days)	Light exposure	Visual cortex	WB of synaptosomes	Immediately after exposure to 0.5, 1, 1.5, or 2 h of a normal-lighted environment	1 h of light exposure is enough to produce an increase of GluN2A, without changes in GluN1 and GluN2B.	[54]
Wistar rats (2 months)	Inhibitory avoidance (step down)	Hippocampus	WB of whole homogenates and synaptic plasma membranes (SPM)	0, 30, and 120 min after training	Increase of GluN1 at 30 min in SPM though not in homogenates; returning to naive and control levels 120 min after training. No changes in GluN2A neither in GluN2B.	[46]
Adult Wistar rats	Inhibitory avoidance (step through)	Hippocampus	WB of whole homogenates and membrane-enriched protein extracts	1 h after training	Increase of GluN1 in membranes, though not in homogenates. No changes in GluN2B.	[55]
Adult Long Evans rats	Single-pellet reaching	Motor cortex	WB of whole homogenates	1 week after training	Increase of GluN1 and GluN2A.	[56]
Wistar rats (2 months)	Open field	Hippocampus	WB of whole homogenates	0, 30, 70 min and 24 hs after training; 0 and 70 min after TEST	Increase of GluN1 and GluN2A, but not GluN2B, at 70 min after training. No changes after TEST.	[34]
Sprague-Dawley rats (10–14 weeks)	Radial arm maze	Frontal cortex and hippocampus	BN PAGE WB of synaptic membranes	6 h after training	Increase of GluN1 and GluN2A in both structures.	[44]
Sprague-Dawley rats (6 days)	Odor preference	Anterior piriform cortex	WB of synaptic membranes	3 h after training	Downregulation of GluN1 surface expression.	[47, 48]
Adult *Neohelice granulata* crabs	Context signal memory	Central brain and thoracic ganglion	WB of BS3-treated homogenates (cross-linking assay)	0, 3, and 24 h after training	Downregulation of GluN1 surface expression in central brain immediately after training; upregulation 3 h later; no difference from naive and controls at 24 h. No changes in the thoracic ganglion.	[43]
Adult Sprague-Dawley rats	Fear conditioning	Hippocampal CA1 region	WB of whole homogenates and synaptic membranes	5–10 min after training	Increase of GluN1 and GluN2B, but not GluN2A, in membranes. No changes in whole CA1 homogenates.	[49]
Wistar rats (1, 2, and 3 months old)	Open field	Hippocampus, amygdala and CPF	WB of whole homogenates	0, 70, 90, 120, 180, and 240 min after training	Transient increase, lasting 90 min or less, of GluN1 and GluN2A, but not of GluN2B 70 min after training, in the hippocampus at all ages. No changes in amygdala neither in CPF at 70 min.	[38]
Wistar rats (3 months old)	Object recognition	Hippocampus and CPF	WB of whole homogenates	0 and 70 min after training and after TEST	Increase of GluN1 and GluN2A, but not GluN2B, 70 min after training in the hippocampus, without changes in CPF. No changes in both structures after TEST.	
